# Real world deployment of a pancreatic cancer risk model: impact of refitting, imputation, and computational burden

**DOI:** 10.1016/j.ebiom.2025.106118

**Published:** 2026-01-08

**Authors:** Wansu Chen, Botao Zhou, Tiffany Q. Luong, Fagen Xie, Bechien U. Wu

**Affiliations:** a Kaiser Permanente Southern California Research and Evaluation, 100 S. Los Robles Ave, Pasadena, CA, USA; b Centre for Pancreatic Care, Department of Gastroenterology, Los Angeles Medical Centre, Southern California Permanente Medical Group, 4867 West Sunset Blvd, Los Angeles, CA, USA

**Keywords:** Risk prediction, Pancreatic cancer, Model deployment, Model refit, Missing predictor imputation

## Abstract

**Background:**

Early detection is a major clinical challenge in pancreatic cancer due to its nonspecific symptoms and frequent late-stage diagnosis. While predictive models using electronic health record (EHR) data show promise, their real world implementation remains underexplored. We previously developed a random survival forest (RSF) model to estimate pancreatic cancer risk using structured EHR data from 2007 to 2017. This study evaluates practical considerations for deploying such a model in a prospective clinical context.

**Methods:**

We refit the original RSF model using a cohort from 2018 to 2019 and evaluated its performance on a 2020 cohort. We assessed how model refitting and different imputation strategies influenced predictive performance and compared execution times to evaluate computational feasibility. Three imputation strategies were tested: sub-model estimation (SME), stacked multiple imputation (SMI), and imputation via fixed chained equations (IFCE). To simulate real time use, we applied the model to 53 sequential weekly patient batches (with average batch size 190,206).

**Findings:**

Refitting improved discrimination and calibration. Without refitting, the C-index ranged from 0.69 to 0.84 depending on imputation method; with refitting, it ranged from 0.79 to 0.83. The IFCE method achieved the best balance between performance (C-index: 0.83 with refit) and runtime (19.54 min). SME had the highest C-index (0.85) and sensitivity (18.41%) but required construction of multiple sub-models. SMI was the most computationally intensive, limiting its scalability in routine use. Calibration improved markedly with refitting. Model performance differed across racial and ethnic groups; calibration was poorest among Black patients but improved with SMI. Execution time varied substantially across methods.

**Interpretation:**

Model refitting and appropriate handling of missing data improve the real world performance of predictive models. Among imputation approaches, IFCE offers the best trade-off between computational efficiency and predictive accuracy. These findings provide practical, implementation-focused guidance for deploying risk prediction models in prospective clinical settings.

**Funding:**

Research reported in this publication was supported by the 10.13039/100000054National Cancer Institute of the 10.13039/100000002National Institutes of Health under Award Number R01CA230442.

**Research in context**

**Evidence before this study**

Several studies have developed prediction models for pancreatic cancer using electronic health record data and showed good discrimination in retrospective analyses. However, very few studies have examined their use in real world clinical settings, and most have not addressed the combined challenges of temporal data drift, missing data at the time of prediction, and computational demands. Prior methodological work has discussed model updating and imputation separately, but rarely in the context of real time prediction for pancreatic cancer.

**Added value of this study**

We prospectively evaluated a previously validated random survival forest model for pancreatic cancer using recent data from over one million patients. We tested whether refitting the model improved predictive performance, compared three different imputation strategies, and measured their impact on discrimination, calibration, and runtime in simulated weekly deployment batches. We also examined model performance across racial and ethnic groups to assess equity implications. This study links model performance, data completeness, and computational feasibility in a real-world setting.

**Implications of all the available evidence**

Our findings show that periodic model refitting improves prediction accuracy, and that the choice of imputation method strongly affects both performance and feasibility. These results provide practical guidance for implementing prediction models in clinical care.

## Introduction

Pancreatic cancer is one of the most lethal malignancies, with a five-year survival rate of 12.8%.^1^ Its poor prognosis is largely due to late diagnosis,^1^ when curative treatment options are limited. Given the absence of population-wide screening programmes and the often subtle, nonspecific symptoms of early disease,^2^^,^^3^ there is growing interest in the use of risk prediction models to identify high-risk individuals for targeted early detection strategies. Recent advances in machine learning and access to large-scale electronic health record (EHR) data have accelerated the development of such models.^4^, ^5^, ^6^, ^7^ These tools can synthesise a wide array of structured clinical data, including demographics, laboratory values, comorbidities, and medications to estimate an individual's risk of developing pancreatic cancer. While numerous models have shown strong predictive performance during validation, the process of translating them into routine clinical use remains underexplored.

In our prior work, we developed and validated three predictive models aimed at early detection of pancreatic cancer using EHR data from 2007 to 2017 with an 18-month follow-up period. The methods included a Cox proportional hazards model, extreme gradient boosting (XGBoost), and a random survival forest (RSF).^6^ While all models demonstrated reasonable performance, the RSF model incorporating age, abdominal pain, weight change, alanine transaminase change and HbA1c as the predictors, showed the most robust discrimination and calibration. It was therefore selected as a high performing representative model to examine key challenges in real world deployment. The purpose of this study is not to re-evaluate modelling approaches, but to investigate practical factors that influence implementation in prospective settings.

Despite their promise, predictive models are infrequently implemented in practice.^8^^,^^9^ One concern is that models developed on historical data may not generalise well when applied prospectively, due to shifts in population characteristics, clinical practice, or data availability. Whether periodic model refitting can mitigate these issues in practice remains an open and context-dependent question. Furthermore, missing data is a pervasive issue in EHRs, and handling incomplete predictor information at the time of prediction is often more difficult than anticipated, particularly in real time deployment.^10^, ^11^, ^12^, ^13^ Lastly, computational considerations such as the complexity of extracting and pre-processing EHR data, computer runtime, and scalability of implementation pipelines often determine whether a model can be realistically integrated into clinical workflow, regardless of its statistical performance.

To address these challenges, we designed a study to systematically evaluate the deployment considerations that arise when applying a previously validated predictive model in a prospective clinical setting. Specifically, we examined whether updating the model with more recent data would improve its predictive performance, how missing data imputation and different imputation strategies might affect implementation accuracy and feasibility, and the computational burden of real world deployment. While previous studies addressed model refitting, imputation, or computational burden in isolation, few have integrated all three together in a prospective deployment simulation. By emulating a prospective screening scenario using sequential patient batches, we aimed to generate practical, evidence-based insights into how predictive models can be effectively translated from development to implementation in routine clinical care. Furthermore, we assessed whether imputation strategy or model refitting differentially affects performance across racial and ethnic groups.

## Methods

### Study setting and data source

This study was conducted within Kaiser Permanente Southern California (KPSC), an integrated healthcare delivery system serving a diverse population of over 4.9 million health plan enrollees. The demographic distribution of study participants reflects the broader KPSC health plan enrollees and the residents of Southern California region.^14^ The EHR system at KPSC (KP HealthConnect) captures comprehensive clinical data across all settings, including outpatient visits, emergency and inpatient encounters, laboratory results, imaging procedures, medication dispensing, and vital signs. Data for this study were extracted from the KPSC Research Data Warehouse (RDW), a centralised data infrastructure that integrates longitudinal EHR, administrative, and claims-based information at the patient level using unique identifiers, as previously described.^15^ The RDW contains mortality files from the National Death Index and can be linked to the institutional tumour registry (which contributes to Surveillance, Epidemiology, and End Results (SEER) Programme) using internal patient identifiers under approved protocols. Demographic variables (e.g., date of birth, sex, race, and ethnicity) are initially self-reported at insurance enrolment and may be updated by patients or their representatives when scheduling appointments or registering for hospital encounters.

### Study design and population

We conducted a retrospective evaluation of a previously validated pancreatic cancer risk prediction model by refitting it using updated EHR data from 2018 to 2020, following its original development on 2007–2017 data. Our analysis emulated prospective deployment to assess how model refitting and imputation strategies influence predictive performance, as well as whether computational demands are feasible.

**Fig. 1:**
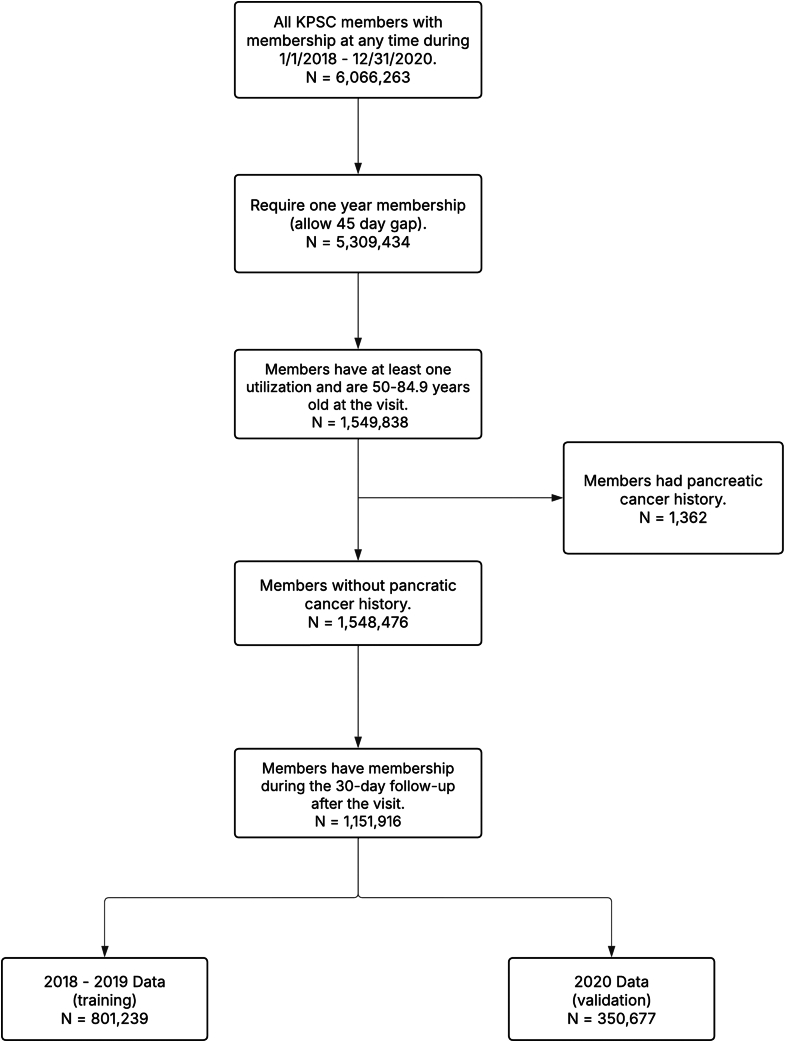
CONSORT flow diagram of cohort construction.

We first constructed a cohort of eligible patients from 2018 through 2020, the latest period for which 18-month follow-up and cancer registry data (available through 2022 at the time of analysis) allowed complete outcome ascertainment (Fig. 1). Eligibility criteria included adults aged 50–84.9 years (selected to match our prior model, minimise competing-risk effects at very old ages, and reflect the target age range recommended by clinical leaders for potential future screening efforts) who had at least one clinic-based visit between 2018 and 2020 (defined as the index date), and continuous health plan enrolment and pharmacy coverage for at least one year prior to that date. Patients with a prior diagnosis of pancreatic cancer were excluded. The inclusion and exclusion criteria used for the original model development^5^ were identically applied to the current cohort. For individuals with multiple qualifying index dates, one was randomly selected to serve as the index date.

This cohort was then split into two subsets. The first subset, comprising individuals with index dates between 2018 and 2019, was used exclusively to refit the previously developed RSF model (refitting cohort). The second subset, consisting of patients with index dates in 2020, was used for model evaluation and prospective batch-based validation (evaluation cohort). Because only one index date was randomly selected per patient, no individual appeared in both subsets. Follow-up lasted up to 18 months from the index date, or until pancreatic cancer diagnosis, death, disenrollment, or the end of the follow-up period, whichever came first.

### Outcome definition

The outcome of interest was incident pancreatic ductal adenocarcinoma (PDAC) within 18 months of the index date, identified using the International Classification of Diseases, Tenth Revision, Clinical Modification (ICD-10-CM) code C25. x and histology codes, as described previously.^5^

### Model refitting approach

The original RSF model was refit using the 2018–2019 refitting cohort. The same predictors identified in the original model development^5^—age, abdominal pain, weight change, change in alanine transaminase (ALT), and HbA1c—were retained for consistency. The RSF model was rerun with these variables to derive updated parameter estimates reflective of the newer dataset. Importantly, hyperparameter values remained consistent with the original development process to ensure comparability, and no additional tuning or cross-validation was performed during this refitting process. This approach allowed us to isolate the impact of temporal updating while preserving the original model framework.

### Missing data imputation strategies

The final model included five predictors: age (continuous; complete), abdominal pain (categorical; complete), and three continuous variables (ALT change, HbA1c, and weight change) which had appreciable missingness; the missingness patterns for all variables are shown in Table 1.

**Table 1:** Characteristics of study subjects at baseline, n (%) unless otherwise stated.

Demographics and lifestyle characteristics	2018–2019 (Training) data N = 801,239	2020 (Validation) data N = 350,677
Age, mean (SD)	64.31 (9.39)	63.12 (9.13)
Female	431,582 (53.86)	185,444 (52.88)
Race/Ethnicity		
Non-Hispanic White	353,199 (44.08)	141,329 (40.30)
Non-Hispanic Black	75,014 (9.36)	32,951 (9.40)
Hispanic	254,790 (31.80)	123,028 (35.08)
Asian and Pacific Islander	94,993 (11.86)	41.001 (11.69)
Multiple/Other/Unknown	23,243 (2.90)	12,368 (3.53)
Family History of Pancreatic Cancer	15,552 (1.94)	6896 (1.97)
Tobacco use		
Ever	282,339 (35.24)	117,650 (33.55)
Never	513,047 (64.03)	229,102 (65.33)
Unknown	5853 (0.73)	3925 (1.12)
Weight Defined by BMI (kg/m^2^)		
Underweight (<18.5)	8528 (1.06)	2659 (0.76)
Normal Weight (18.5–24.9)	167,572 (20.91)	60,544 (17.26)
Overweight (25–29.9)	257,438 (32.13)	99,237 (28.30)
Obese (30+)	271,442 (33.88)	112,204 (32.00)
Unknown	96,259 (12.01)	76,033 (21.68)
Weight Change in 1 Year in lb., median (IQR)	0.00 (−0.02, 0.02)	−0.00 (−0.03, 0.02)
**Lab tests**		
ALT, IU/L		
N	375,354	154,237
Most recent, median (IQR)	22.00 (17.00, 29.00)	22.00 (17.00, 30.00)
Change in 1 year, median (IQR)	0.00 (−4.00, 4.00)	0.00 (−4.00, 4.00)
Change rate, median (IQR)	0.00 (−0.01, 0.01)	0.00 (−0.01, 0.01)
HgA1c, %		
N	473,164	187,249
Most recent, median (IQR)	5.90 (5.50, 6.60)	5.90 (5.50, 6.80)
Change in 1 year, median (IQR)	−0.10 (−0.40, 0.20)	0.00 (−0.20, 0.20)
HGB for Males, g/dL		
N	193,214	80,508
Most recent, median (IQR)	14.60 (13.40, 15.40)	14.60 (13.50, 15.50)
HGB for Females, g/dL		
N	231,890	93,559
Most recent, median (IQR)	13.30 (12.40, 14.10)	13.30 (12.40, 14.10)
HCT, L/L		
N	425,102	174,066
Most recent, median (IQR)	41.50 (38.50, 44.20)	41.90 (39.00, 44.70)
Change in 1 year, median (IQR)	0.00 (−1.80, 1.80)	−0.10 (−2.00, 1.70)
RBC, million/mm^3^		
N	412,591	168,256
Most recent, median (IQR)	4.57 (4.23, 4.90)	4.59 (4.25, 4.93)
Total Cholesterol, mg/dL		
N	380,534	148,892
Most recent, median (IQR)	179 (149, 210)	179.00 (148, 211)
Platelets, count/L		
N	205,332	82,303
Change rate in 1 year, median (IQR)	0.01 (−0.05, 0.07)	0.00 (−0.06, 0.06)
**Medical Conditions, 0–6 month prior**		
Acute Pancreatitis	1487 (0.19)	606 (0.17)
Chronic Pancreatitis	831 (0.10)	346 (0.10)
Benign Pancreatic Disease	2086 (0.26)	931 (0.27)
**Medical Procedures, 0–6 month prior**		
Surgical Procedures on oesophagus	6331 (0.79)	2424 (0.69)
**Medications, 0–6 month prior**		
Pancreatic Enzyme	588 (0.07)	268 (0.08)
**GI-Related Signs/Symptoms, 0–6 month prior**		
Abdominal Pain	65,625 (8.19)	28,810 (8.22)
Constipation	27,699 (3.46)	10,649 (3.04)
Melaena	4072 (0.51)	1589 (0.45)

We evaluated the impact of handling missing predictor values using three distinct strategies. Sub-model estimation (SME) generates predictions using sub-models developed based solely on observed (non-missing) predictor values.^12^^,^^16^ Stacked multiple imputation (SMI) imputes missing values in new patient data by combining these data with an existing development dataset, thereby leveraging information from the larger pooled dataset.^17^ For SME, because only three predictors exhibited appreciable missingness (ALT change, HbA1c, and weight change), we developed eight sub-models (2^3^ combinations) corresponding to all possible observed–missing patterns, as illustrated in the “8 scenarios” section of the SME code in Appendix II. Imputation via fixed chained equations (IFCE) uses a predefined, sequential approach in which missing values are imputed using pre-estimated models in a fixed order.^12^ Unlike SMI, IFCE does not require access to the original development dataset at the time of imputation and instead relies on pre-trained, variable-specific imputation models.

Each strategy was implemented with both the original and refit models, enabling direct comparisons across modelling and imputation configurations. Additional details regarding the imputation procedures and code implementations are provided in Appendix I and Appendix II of the online Supplementary Materials, respectively.

SMI requires access to the original development dataset at the time of imputation to enable stacking of incoming cases with the development data. In contrast, IFCE can be applied without access to the development dataset at imputation time because it relies on pre-estimated, variable-specific imputation models, and SME can be applied provided the corresponding set of missingness-pattern sub-models is available.

### Computational efficiency

We also assessed computational efficiency by comparing data extraction time (in hours) and imputation and model execution time (in minutes) across the three imputation strategies. This evaluation ensured that runtime burden was comparable and clinically feasible. All imputation strategies were applied in conjunction with both the original and refitted models, allowing for direct comparisons across combinations of modelling and imputation approaches.

### Model evaluation metrics

Model performance was assessed using the 2020 evaluation cohort. Discrimination was measured using Harrell's concordance index (C-index). We also evaluated sensitivity, positive predictive value (PPV), and number needed to screen at clinically relevant risk thresholds (top 1% and 2.5% predicted risk). Calibration was assessed by calibration plots with five risk groups (<50th, 50–74th, 75–89th, 90–94th, 95–100th percentiles). Greenwood-Nam-D’Agostino (GND) calibration test was also performed to assess goodness-of-fit.^16^

### Simulated prospective deployment

To reflect the dynamics of rolling model deployment, we simulated 53 weekly sequential batches. For each batch, data processing, imputation, and model execution were carried out independently, mimicking what would occur in a real time implementation pipeline.

To assess the feasibility of model implementation, we recorded the time required for key computational steps, including data extraction and preparation, imputation, and risk score generation. Execution times were averaged across all batches and stratified by imputation method to provide practical insights into resource demands. The code is provided in Appendix II of the online Supplementary document.

### Model performance by race and ethnicity

To assess whether model refitting and imputation strategy differentially affect performance across racial and ethnic groups, we conducted stratified analyses of model discrimination and calibration across four mutually exclusive groups: Non-Hispanic White, Black, Hispanic, and Asian and Pacific Islander. The same evaluation metrics mentioned above were applied.

### Computing environment

All analyses were conducted within a secure, high performance Linux-based computing environment maintained by our institution. The SAS system was equipped with a total of 9 servers with 256 GB of RAM per node and 32 cores. The R server consists of one single node with 36 cores (hyperthreaded to 72 threads), 1 TB of RAM, and an NVIDIA A100 GPU. It utilised SAN-backed, expandable storage and dual 10 GbE network interfaces configured with LACP bonding for high throughput and redundancy. Analytical workflows were implemented using R (version 4.3.0) and SAS (version 9.4). Modelling and imputation routines were executed using established R packages, including randomForestSRC for RSF modelling, missRanger for SMI and mice for IFCE. Parallel processing capabilities on the SAS server were not utilised.

### Role of the Funding Source

The funders of the study had no role in the study design, data collection, analysis, data interpretation, or manuscript preparation.

### Ethics

This study protocol was reviewed and approved by the KPSC Institutional Review Board (IRB# 12135). The requirement for informed consent was waived by the IRB because the study used deidentified data and posed minimal risk to participants. All study procedures complied with the Declaration of Helsinki and all applicable institutional and federal ethical regulations. The authors did not use any AI or AI-assisted technologies in the writing process.

## Results

### Study cohorts and baseline characteristics

A total of 801,239 patients from 2018 to 2019 were included in the refitting cohort, and 350,677 patients from 2020 comprised the evaluation cohort. Baseline demographic and clinical characteristics are summarised in Table 1. Patients in the evaluation cohort had a slightly lower mean age compared to the refitting cohort (63.1 vs. 64.3 years), and the proportion of females was similar across cohorts (52.9% vs. 53.9%).

Both the refitting and evaluation cohorts were predominantly non-Hispanic White and Hispanic, followed by Asian and Pacific Islander and non-Hispanic Black patients, with similar distributions across the two cohorts (e.g., 44.1% vs. 40.3% non-Hispanic White and 31.8% vs. 35.1% Hispanic, respectively).

Patient characteristics stratified by race and ethnicity are provided in Table E1. Notable differences were observed across groups in demographics, lifestyle factors (e.g., tobacco use and BMI), and family history of pancreatic cancer.

### Original vs. refit models

Refitting the RSF model using the 2018–2019 cohort led to consistent improvements in predictive performance across all modelling configurations when evaluated on the 2020 evaluation cohort (Table 2). Without refitting, discrimination as measured by the C-index ranged from 0.69 to 0.84, depending on whether imputation was applied and the imputation method used. After refitting, the C-index improved across all scenarios, ranging from 0.79 to 0.85, with the most notable gain in the non-imputed condition (from 0.69 to 0.81). Similarly, the SME, SMI, and IFCE methods all demonstrated improved discrimination following refitting (Table 2).

**Table 2:** Model performance by imputation method and model refitting status.

	Without refit				With refit			
	Without imputation	With imputation			Without imputation	With imputation		
		SME^a^	SMI^b^	IFCE^c^		SME^a^	SMI^b^	IFCE^c^
N	40,300	350,677	350,677	350,677	40,300	350,677	350,677	350,677
All PDAC cases	49	253	253	253	49	253	253	253
C-index	0.69	0.84	0.75	0.81	0.81	0.85	0.79	0.83
At top 1% risk								
Sensitivity, %	10.2	18.18	9.88	14.62	18.37	23.72	13.44	20.55
PPV, %	1.52	1.60	0.87	1.29	2.73	2.09	1.18	1.81
Cases detected	5	46	25	37	9	60	34	52
Patients screened	330	2877	2884	2877	330	2877	2877	2877
Screened per case	66	63	115	78	37	48	85	55
At top 2.5% risk								
Sensitivity, %	12.24	28.46	18.58	26.88	26.53	29.64	24.11	30.83
PPV, %	0.73	1.00	0.65	0.95	1.58	1.04	0.85	1.08
Cases detected	6	72	47	68	13	75	61	78
Patients screened	824	7191	7194	7191	824	7191	7194	7191
Screened per case	137	100	154	105	63	96	118	93

a **SME**: Sub-models estimation.

b **SMI**: Stacked multiple imputation.

c **IFCE**: Imputation by fixed chained equations.

**Fig. 2:**
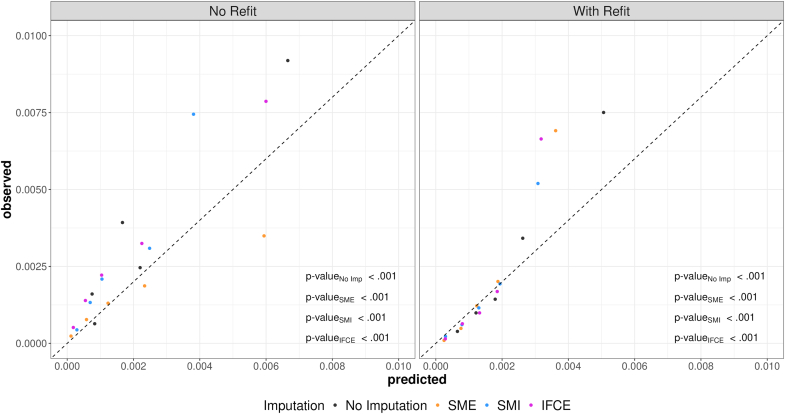
Calibration plots. Left: No refit; Right: With Refit. X-axis: predicted; Y-axis: observed. The five clusters represent the five risk groups defined by the ranges of predicted risks: <50th, 50–74th, 75–89th, 90–94th, 95–100th percentiles. Black: SME; Orange: SMI; Blue: IFCE.

Refitting improved alignment between predicted and observed risks across most groups, with residual miscalibration primarily confined to the 95–100th percentile (GND p < 0.001 across all methods), as shown in Fig. 2.

### Impact of imputation on model eligibility

In the absence of imputation, only a small fraction of the evaluation cohort had complete data for all model predictors, approximately one-eighth the size of the cohort eligible for prediction when imputation was applied. However, among this reduced set of complete cases, model performance was markedly stronger. Using the refit model without imputation, the PPV in the top 1% risk group reached 2.7%, substantially higher than PPV estimates observed with any of the imputation strategies.

### Imputation strategy comparisons

Different imputation strategies had varying effects on both model performance and computational feasibility. The SME approach yielded the highest PPV and sensitivity at the top 2.5% threshold (e.g., sensitivity: 23.72% with refitting), indicating strong ability to detect true cases within a limited screening population.

Among the evaluated methods, IFCE demonstrated a favourable balance between predictive accuracy and runtime efficiency. With the refit model, IFCE achieved a C-index of 0.83 and provided reasonable sensitivity and PPV at both the top 1% and 2.5% risk thresholds (Table 2), with modest computational demands. Although SMI showed slightly better calibration in some scenarios (Fig. 2), it exhibited the poorest discrimination and was the most computationally intensive method (Table 2).

### Simulation of real world prospective deployment

In the prospective simulation using the evaluation cohort, 53 weekly batches were formed, each averaging 190,206 individuals. The SME approach achieved the highest average C-index (0.85) and consistently outperformed other methods in capturing future pancreatic cancer cases within the top risk strata. For example, when using SME with the refit model, 28.63% of future pancreatic cancer cases were identified within the top 2.5% risk group, indicating substantial enrichment of true positives in a small high-risk population. The IFCE method also demonstrated strong performance with practical feasibility, requiring a mean runtime of 19.54 min per batch while capturing 22.91% of cases in the top 2.5% risk group. In contrast, SMI was not only less effective in prediction but also computationally intensive, requiring an average of over 6 h per batch (383.77 min), which may limit scalability. These performance comparisons are summarised in Table 3.

**Table 3:** Summary of simulation results, data extraction time, imputation time and model execution time.

	SME	SMI	IFCE
Data extraction time (hours)	13.30	13.30	13.30
Imputation and model execution time (minutes)	0.71	383.77	19.54
C-index	0.85	0.76	0.79
At top 1% risk			
Sensitivity, %	18.41	10.89	15.79
PPV, %	1.72	1.00	1.48
Cases detected	28	17	24
Patients screened	1648	1676	1646
Screened per case	60	104	73
At top 2.5% risk			
Sensitivity, %	28.63	17.28	24.27
PPV, %	1.07	0.63	0.91
Cases detected	43	26	37
Patients screened	4114	4190	4113
Screened per case	95	163	116

Based on an average of 53 simulated cohorts with 190,206 eligible patients. In the 18 months after the index date, 152 patients developed PDAC.

Batch-level cohort characteristics and processing times are presented in Table E2. Detailed batch-level predictive performance metrics, including C-index, sensitivity, PPV, and runtime, are reported in Tables E3a to c for each imputation strategy: SME (Table E3a), SMI (Table E3b), and IFCE (Table E3c). These supplemental tables provide additional insight into the variability and computational consistency of model deployment over time.

### Model performance by race and ethnicity

**Fig. 3:**
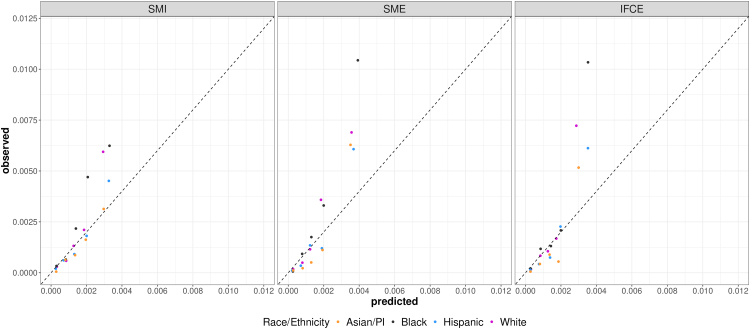
Calibration plots with refit model by race and ethnicity. Left: SMI; Middle: SME; Right: IFCE. X-axis: predicted; Y-axis: observed. The five clusters represent the five risk groups defined by the ranges of predicted risks: <50th, 50–74th, 75–89th, 90–94th, 95–100th percentiles. Orange: Asian/Pacific Islander patients; Black: Black patients; Blue: Hispanic patients; Red: Non-Hispanic White patients.

Model performance varied by imputation method and race/ethnicity, with C-index values ranging from 0.78 to 0.89 across groups (Table 4). Sensitivity was highest among Asian/Pacific Islander patients using SME (33.33%) at the top 1% risk threshold and IFCE (42.86%) at the top 2.5% risk threshold, while PPV values were consistently higher in Black patients. SME approach continued to perform well in terms of sensitivity and PPV across all the racial and ethnic groups (Table 4). Calibration is poorest for Black patients, but SMI was the best among all three imputation strategies (Fig. 3).

**Table 4:** Model performance by imputation method and race/ethnicity based on the refit model.

	SME				SMI				IFCE			
	White	Black	Hispanic	Asian/PI	White	Black	Hispanic	Asian/PI	White	Black	Hispanic	Asian/PI
C-index	0.84	0.87	0.85	0.89	0.78	0.80	0.79	0.82	0.81	0.82	0.83	0.87
At top 1% risk												
Sensitivity, %	24.35	25.00	16.46	33.33	13.04	13.89	15.19	14.29	21.74	16.67	21.52	28.57
PPV, %	1.98	2.73	1.06	1.70	1.06	1.52	0.97	0.73	1.77	1.82	1.38	1.46
Cases detected	28	9	13	7	15	5	12	3	25	6	17	6
Patients screened	1414	330	1231	411	1414	330	1231	411	1414	330	1231	411
Screened per case	51	37	94	59	94	66	103	137	56	55	72	68
At top 2.5% risk												
Sensitivity, %	26.96	36.11	25.32	38.10	24.35	19.44	18.99	23.81	30.43	25.00	27.85	42.86
PPV, %	0.88	1.58	0.65	0.78	0.79	0.85	0.49	0.49	0.99	1.09	0.72	0.88
Cases detected	31	13	20	8	28	7	15	5	35	9	22	9
Patients screened	3534	824	3076	1026	3534	824	3076	1026	3534	824	3076	1026
Screened per case	114	63	154	128	127	118	204	204	101	92	139	114

## Discussion

This study provides a comprehensive assessment of key considerations for applying a previously validated pancreatic cancer risk prediction model in a real world setting. We found that refitting the original RSF model substantially improved both discrimination and calibration when applied prospectively to a new cohort. A major strength of this study lies in its focus on practical model application, which remains underrepresented in the prediction modelling literature. By leveraging recent EHR data and simulating deployment across 53 weekly patient batches, we evaluated not only statistical performance but also computational efficiency, factors critical to successful integration in clinical workflows.

Refitting the model using a more recent cohort consistently improved predictive performance across all configurations. These results reinforce existing evidence that periodic recalibration is necessary to maintain model validity over time.^17^, ^18^, ^19^ Prior studies have shown that prediction models often degrade in performance due to temporal shifts in patient characteristics, clinical practice, and healthcare delivery.^20^, ^21^, ^22^ Given the increasing adoption of prediction models in clinical practice, establishing processes for ongoing updating and validation will be essential to maintaining long-term prediction accuracy.

Our evaluation of imputation strategies highlighted important trade-offs among predictive performance, population coverage, and computational feasibility. Complete-case analysis (i.e., no imputation) yielded the highest PPV but limited predictions to a small subset of patients with complete data. These individuals likely had more frequent healthcare interactions and richer data, potentially indicating higher actual risk, but also introducing selection bias. In contrast, imputation-based methods enabled risk estimation across a much larger population, substantially expanding the potential reach of screening or early detection efforts.

Among the imputation methods, the SME approach yielded the highest PPV and sensitivity at relevant risk thresholds but required generating custom models for each pattern of missingness, posing scalability challenges for prospective deployment. SMI, while offering modest calibration improvements in select scenarios, was the most computationally demanding and may be impractical for real time or high-frequency use. In contrast, IFCE struck a practical balance, delivering strong predictive performance with acceptable runtime burden. These findings align with prior research indicating that while complex or highly accurate imputation methods can be appealing from a statistical perspective, they often impose resource demands that limit their utility in real world clinical environments.^23^

Evaluating model performance in weekly batch-based simulations allowed us to observe how predictive and computational metrics evolve over time. The SME method continued to yield the highest discrimination and case capture within high-risk strata across these simulations, although its computational intensity remains a barrier. IFCE, with a mean execution time of less than 20 min per batch, showed promising balance and scalability, especially for systems aiming to screen large populations without specialised infrastructure. These simulations underscore the importance of aligning modelling decisions not only with statistical goals, but also with the logistical constraints in real world informatics environments.

The observed underestimation of risk in the highest predicted risk group, even after refitting, may reflect differences in healthcare utilisation patterns between the model's training and evaluation periods. The refit model was trained on data from 2018 to 2019, with follow-up during the acute phase of the COVID-19 pandemic—a period marked by reduced healthcare access and delayed diagnoses. In contrast, the 2020 evaluation cohort experienced follow-up during a rebound phase. This temporal misalignment may have led to increased case capture post-COVID, causing underestimation in the highest risk strata. These findings emphasise the need for careful attention to healthcare delivery trends when interpreting model calibration.

Differences in model performance across racial and ethnic groups highlight the importance of equity-focused evaluation. Calibration was poorest for Black patients, consistent with our prior findings in the original development dataset (2007–2017), where calibration was poorer yet PPV was higher due to elevated disease incidence in this group.^24^ This pattern is not unexpected: because the model was trained on the pooled population without subgroup-specific recalibration, the higher baseline incidence among Black patients likely contributed to systematic risk underestimation, which could reduce their likelihood of being flagged for further evaluation. Although the SMI approach improved calibration somewhat for Black patients, SME consistently achieved higher PPV and sensitivity across all groups. These findings underscore the need for proactive safeguards, such as subgroup-specific recalibration, prospective equity audits, and engagement of stakeholders from affected communities, to ensure that predictive tools promote, rather than hinder, equity in early cancer detection. Race and ethnicity are treated here as proxies for social and structural factors, not biological variables.

This study evaluated a single risk assessment per patient and did not examine longitudinal re-scoring. We made this design choice because the appropriate cadence for reassessing risk is inherently programme-specific, depending on the intended clinical application, the outcome window, local workflows, and the scope and intensity of downstream interventions. In practice, programmes may define a minimum interval (“reassessment window”) during which patients are not eligible for repeat scoring, such as every 3, 6, or 12 months. This decision has important operational implications: shorter intervals would increase the number of eligible patients in each cycle and therefore the computational burden, whereas longer intervals would reduce this burden but delay detection of newly high-risk individuals. Because these parameters must be defined by individual programmes, their impact on performance and resource demand cannot be generalised and was therefore beyond the scope of the current study. Our findings on per-batch runtime can help inform planning, but the cadence of reassessment should be determined locally based on programme goals and capacity.

Although we report detailed runtime estimates for data extraction, imputation, and model execution, it is important to emphasise that these results reflect our institution's computing environment. Execution times are influenced by hardware specifications, system load, and technical infrastructure, including whether operations are multi-threaded or rely on shared memory or disk I/O. Institutions using different technical environments may experience different runtime characteristics and should plan accordingly. Furthermore, implementation success depends not only on computational logistics but also on human factors, including clinical usability, staff training, and the availability of resources for downstream diagnostic evaluation. These elements were beyond the scope of the current study but represent critical next steps in translating model-based risk prediction into actionable clinical programmes.

Additional limitations must be acknowledged. This study was conducted within a single integrated healthcare system, which may limit generalisability to other settings with different populations, care models, or data capture practices. While we examined three imputation strategies, we did not explore deep learning or federated methods which may be suitable in more complex or privacy-sensitive contexts. Lastly, our simulations treated each batch as independent and did not model longitudinal risk reassessments. In real world settings, patients may be flagged as high-risk repeatedly over time, which could influence clinical decisions and outreach strategies. Future work should examine how dynamic risk updates integrate into longitudinal care.

For future implementation in other populations, refitting the model on local data by re-estimating the baseline hazard and updating model coefficients using the same predictors may help improve calibration while preserving discrimination. We recommend reassessing calibration after refitting using risk-group plots and Greenwood–Nam–D’Agostino statistics, as demonstrated in this study.

### Conclusion

In this prospective evaluation of a pancreatic cancer risk prediction model, we demonstrate that model refitting and thoughtful handling of missing data are essential for sustaining predictive performance in real world settings. These findings underscore the importance of aligning modelling decisions with implementation realities, balancing statistical rigour with usability. As predictive analytics move from theory to practice, such considerations will be critical to their long-term success.

## Contributors

Wansu Chen: Conceptualisation, Methodology, Validation, Investigation, Writing–Original Draft, Writing–Review & Editing, Visualisation, Supervision; Botao Zhou: Methodology, Software, Validation, Formal analysis, Investigation, Data Curation, Writing–Review & Editing, Visualisation; Tiffany Luong: Writing–Review & Editing, Project administration; Fagen Xie: Methodology, Data Curation, Writing–Review & Editing; Bechien Wu: Conceptualisation, Methodology, Validation, Writing–Review & Editing, Supervision, Funding acquisition. All authors have approved the final submitted draft.

## Data sharing statement

Anonymised data supporting this study's findings may be shared upon reasonable request, contingent on: (1) collaboration with the study team on all resulting publications, (2) provision of funding to cover administrative and investigator time, (3) proof of relevant qualifications and human subjects protections training, and (4) execution of data use agreements between institutions.

## Code sharing

The code is included in the supplementary material.

## Declaration of interests

We declare no competing interests.
