# Supplementary material for: Real world deployment of a pancreatic cancer risk model: impact of refitting, imputation, and computational burden
Source: eBioMedicine. 2026 Jan 8;124:106118. doi: 10.1016/j.ebiom.2025.106118 (PMC12813562; doi:10.1016/j.ebiom.2025.106118)
Supplement: Appendix [file mmc1.docx]

**Appendix I. Missing Data Imputation Strategies**

For all three methods below, outcome variable is assumed unknown for all new patients for whose predictor(s) needs to be imputed.

**Sub-model estimation (SME):**

Sub-model estimation (SME) is an imputation method that focuses on developing prediction models using only the observed (non-missing) data for specific predictor combinations. When a predictor is missing, SME generates a sub-model using only the available predictors for that specific combination. For example, if a model has two predictors, X_1_ and X_2_, but X_1_ is missing, a sub-model is developed using only X_2_. Similarly, if X_2_ is missing, a separate sub-model using X_1_ is created. This approach allows for flexible and efficient handling of missing data without requiring a complete dataset. The number of sub-models grows exponentially with the number of predictors, as each possible combination of missing and observed values requires a separate model. For k predictors, this results in 2^k^ possible sub-models. These sub-models are then used to generate predictions for patients with missing predictor values.

SME offers several advantages. It can efficiently handle missing data without the need for a complete dataset and provides flexibility, allowing the model to generate predictions even when some predictors are missing. This reduces the computational burden by avoiding the complexity of developing a single full model that can account for all possible combinations of missing predictors. However, the method also has some limitations. The exponential growth in the number of sub-models can become impractical for high-dimensional data with many predictors. Additionally, each sub-model must be carefully validated to ensure that it provides accurate predictions. Finally, SME assumes that the relationship between predictors and outcomes can be adequately captured by the various sub-models, which may not always be the case.

**Stacked multiple imputation (SMI):**

Stacked Multiple Imputation (SMI) is an imputation method that addresses missing data by combining new patient data (one or more records) with an existing, pre-developed dataset, creating a combined dataset for the imputation process. This combined dataset is then used to generate imputed values for missing predictors in the new patient data. The method leverages the information available in the original development dataset to inform the imputation, ensuring that the imputed values are consistent with the patterns and relationships observed in the larger data.

One of the primary advantages of SMI is that it can handle multiple missing values for each new patient without requiring a complete model re-estimation. The method can efficiently use the structure of the original dataset to produce reliable imputations. This approach minimizes the need for developing new imputation models each time new patient data is encountered, which can be particularly useful in prospective or real-time applications. However, SMI has some important considerations. Because it relies on the original development dataset, the imputation process may involve significant computational overhead, especially if the development dataset is large. To mitigate this, it is recommended to pre-derive and store the imputation models using the development data, allowing for faster imputation of new patient data without having to recompute the models each time. At the time of implementation, first load the pre-derived imputation models, append new cases to the development dataset, and apply the pre-derived imputation models. Once the missing values in the new patient data are imputed, one can separate these new cases from the combined dataset for analysis or further processing. In the current study, we retrained imputation models every time we appended new cases instead of using pre-derived models.

In summary, SMI offers a practical and efficient approach to imputing missing data in new patients, leveraging the strength of an existing development dataset without the need for constant model recalibration. However, its reliance on a large dataset and the associated computational cost must be carefully managed.

Stacked multiple imputation (SMI) involves combining the new patient data (1 or more records) with the original development dataset and performing imputation on the entire combined dataset. This method does not require access to the imputation model parameters, and it can handle multiple missing values in the new patient. However, this approach is associated with the increased computational burden because the imputation process involves the entire development dataset. In addition, it requires access to the entire development dataset, which may not be feasible in reality. To avoid repeating the imputation process each time a new patient is added, it is suggested that pre-deriving and pre-storing the imputed development data. This approach maintains the separation between the development data and new cases, ensuring that the imputation models are not influenced by the new patients and the computational resource is preserved.

**Imputation by fixed chained equations (IFCE):**

Imputation by Fixed Chain Equations (IFCE) is an imputation method that uses a sequential and deterministic approach to fill in missing values based on a predefined, fixed sequence of equations. Unlike traditional multiple imputation by chained equations (MICE), which involves iterative and stochastic modeling of missing values, IFCE maintains a consistent order and fixed model structure, ensuring that the imputation process is both transparent and reproducible. The process of IFCE begins with the establishment of a fixed chain, which is a predetermined sequence of models used for imputing each variable. For example, if a dataset has three variables X1, X2, and X3, the imputation process might specify that X1 is imputed first using the available values of X2 and X3. Next, X2 is imputed using the observed value of X3, and the (observed + imputed) value of X1. Finally, X3 is imputed using the (observed + imputed) values of X1 and X2. Each variable is imputed using a model that is consistent across all imputations, ensuring that the process is deterministic. The outcome variable, which is also missing for new patients in any prospective implementation, is imputed during this process by default but should be discarded, as it is not needed for predictions.

One key advantage of IFCE is that it does not require access to the original development dataset at the time of imputation. Once the models for each variable in the fixed chain are derived and stored, they can be applied directly to any new data. This makes IFCE highly efficient, as the pre-trained models can be quickly loaded and applied without recalculating or retraining. The process is therefore scalable and suitable for real-time or high-throughput applications, such as clinical decision support or prospective model implementation. However, IFCE has some limitations. Since the imputation models are fixed and predetermined, they may not adapt well to data with complex or highly variable patterns. The accuracy of the imputed values depends directly on the quality and relevance of the pre-specified models. Additionally, IFCE can become less effective when the relationships between variables are highly nonlinear or when the missing data pattern is complex.

In summary, IFCE is a deterministic, rule-based imputation method that provides fast and consistent imputations by following a fixed chain of equations. It is well-suited for situations where transparency, reproducibility, and computational efficiency are priorities. Crucially, IFCE does not require access to the original development dataset at the time of imputation. Only the pre-trained models are needed.

**References:**

1. Marshall G, Warner B, MaWhinney S, Hammermeister K. Prospective prediction in the presence of missing data. *Stat Med*. 2002; **21**(4): 561-570. <https://doi.org/10.1002/sim.966>.
2. Janssen KJM, Vergouwe Y, Donders ART, et al. Dealing with missing predictor values when applying clinical prediction models. *Clin Chem*. 2009; **55**(5): 994-1001. <https://doi.org/10.1373/clinchem.2008.115345>.
3. Hoogland, J., van Barreveld, M., Debray, T. P., Reitsma, J. B., Verstraelen, T. E., Dijkgraaf, M. G., & Zwinderman, A. H. (2020). Handling missing predictor values when validating and applying a prediction model to new patients. *Statistics in medicine*, *39*(25), 3591-3607.

**Appendix II. Programming Codes for Data Imputation and Model Implementation**

library(dplyr)

library(doParallel)

library(haven)

library(survival)

library(randomForestSRC)

set.seed(1234)

# read cohort data

cohort_data <- read_sas(".../cohort_data.sas7bdat")

################################################################################

## SME ##

################################################################################

# Because SME was applied only to the three predictors with appreciable missingness (ALT change, #HbA1c, and weight change), 8 sub-models (2³ combinations) were developed—one for each #observed/missing pattern (“8 scenarios”) as shown below.

# 8 scenarios:

# ALT HGBA1C Wight Change

# 1 0 0 0

# 2 1 0 0

# 3 1 1 0

# 4 1 0 1

# 5 1 1 0

# 6 1 0 1

# 7 0 1 1

# 8 1 1 1

cohort_data_1 <- cohort_data[is.na(cohort_data$ALT_result_change) &

is.na(cohort_data$HGBA1C_result_most_recent) &

is.na(cohort_data$weight_change_per_day),]

cohort_data_2 <- cohort_data[!is.na(cohort_data$ALT_result_change) &

is.na(cohort_data$HGBA1C_result_most_recent) &

is.na(cohort_data$weight_change_per_day), ]

cohort_data_3 <- cohort_data[is.na(cohort_data$ALT_result_change) &

!is.na(cohort_data$HGBA1C_result_most_recent) &

is.na(cohort_data$weight_change_per_day), ]

cohort_data_4 <- cohort_data[is.na(cohort_data$ALT_result_change) &

is.na(cohort_data$HGBA1C_result_most_recent) &

!is.na(cohort_data$weight_change_per_day), ]

cohort_data_5 <- cohort_data[!is.na(cohort_data$ALT_result_change) &

!is.na(cohort_data$HGBA1C_result_most_recent) &

is.na(cohort_data$weight_change_per_day), ]

cohort_data_6 <- cohort_data[!is.na(cohort_data$ALT_result_change) &

is.na(cohort_data$HGBA1C_result_most_recent) &

!is.na(cohort_data$weight_change_per_day), ]

cohort_data_7 <- cohort_data[is.na(cohort_data$ALT_result_change) &

!is.na(cohort_data$HGBA1C_result_most_recent) &

!is.na(cohort_data$weight_change_per_day), ]

cohort_data_8 <- cohort_data[!is.na(cohort_data$ALT_result_change) &

!is.na(cohort_data$HGBA1C_result_most_recent) &

!is.na(cohort_data$weight_change_per_day), ]

# Refit 8 sub-models

rsf_tree1 <- rfsrc(Surv(daysfu, pdac_18mos) ~ .,

data = training_data_1,

ntree=50, nodedepth =6, mtry = 5, nodesize = 150,

importance = FALSE, tree.err=FALSE)

rsf_tree2 <- rfsrc(Surv(daysfu, pdac_18mos) ~ .,

data = training_data_2,

ntree=50, nodedepth =6, mtry = 5, nodesize = 150,

importance = FALSE, tree.err=FALSE)

rsf_tree3 <- rfsrc(Surv(daysfu, pdac_18mos) ~ .,

data = training_data_3,

ntree=50, nodedepth =6, mtry = 5, nodesize = 150,

importance = FALSE, tree.err=FALSE)

rsf_tree4 <- rfsrc(Surv(daysfu, pdac_18mos) ~ .,

data = training_data_4,

ntree=50, nodedepth =6, mtry = 5, nodesize = 150,

importance = FALSE, tree.err=FALSE)

rsf_tree5 <- rfsrc(Surv(daysfu, pdac_18mos) ~ .,

data = training_data_5,

ntree=50, nodedepth =6, mtry = 5, nodesize = 150,

importance = FALSE, tree.err=FALSE)

rsf_tree6 <- rfsrc(Surv(daysfu, pdac_18mos) ~ .,

data = training_data_6,

ntree=50, nodedepth =6, mtry = 5, nodesize = 150,

importance = FALSE, tree.err=FALSE)

rsf_tree7 <- rfsrc(Surv(daysfu, pdac_18mos) ~ .,

data = training_data_7,

ntree=50, nodedepth =6, mtry = 5, nodesize = 150,

importance = FALSE, tree.err=FALSE)

rsf_tree8 <- rfsrc(Surv(daysfu, pdac_18mos) ~ .,

data = training_data_8,

ntree=50, nodedepth =6, mtry = 5, nodesize = 150,

importance = FALSE, tree.err=FALSE)

################################################################################

## SMI ##

################################################################################

# read previous (2007-2017) data

prev_cohort <- read_sas(".../prev_cohort.sas7bdat")

prev_cohort$source = 'prev'

# combine two cohorts

cohort_data_2 <- cohort_data

cohort_data_2$source = 'new'

combo_data <- rbind(prev_cohort, cohort_data_2)

# imputation

library(missRanger)

imp_data <- missRanger(combo_data,

num.trees = 100,

num.threads = 20,

pmm.k = 5,

returnOOB = FALSE)

# Refit model based on impuation data

cohort_data_SMI <- imp_data[which(imp_data$source == "new"),]

rsf_tree_SMI <- rfsrc(Surv(daysfu, pdac_18mos) ~ .,

data = cohort_data_SMI,

ntree=50, nodedepth =6, mtry = 5, nodesize = 150,

importance = FALSE, tree.err=FALSE)

################################################################################

## IFCE ##

################################################################################

library(mice)

# imputation

data_mice <- mice(cohort_data, m=5, maxit=10, print=FALSE)

data_imp <- complete(data_mice, action = "long", include = TRUE)

data_1 <- data_imp[data_imp$.imp == 1,]

data_2 <- data_imp[data_imp$.imp == 2,]

data_3 <- data_imp[data_imp$.imp == 3,]

data_4 <- data_imp[data_imp$.imp == 4,]

data_5 <- data_imp[data_imp$.imp == 5,]

# refit 5 rsf models one for each dataset

rsf1 <- rfsrc(Surv(daysfu, pdac_18mos) ~ .,

data = data_1,

ntree=50, nodedepth =6, mtry = 5, nodesize = 150,

importance = FALSE, tree.err=FALSE)

rsf2 <- rfsrc(Surv(daysfu, pdac_18mos) ~ .,

data = data_2,

ntree=50, nodedepth =6, mtry = 5, nodesize = 150,

importance = FALSE, tree.err=FALSE)

rsf3 <- rfsrc(Surv(daysfu, pdac_18mos) ~ .,

data = data_3,

ntree=50, nodedepth =6, mtry = 5, nodesize = 150,

importance = FALSE, tree.err=FALSE)

rsf4 <- rfsrc(Surv(daysfu, pdac_18mos) ~ .,

data = data_4,

ntree=50, nodedepth =6, mtry = 5, nodesize = 150,

importance = FALSE, tree.err=FALSE)

rsf5 <- rfsrc(Surv(daysfu, pdac_18mos) ~ .,

data = data_5,

ntree=50, nodedepth =6, mtry = 5, nodesize = 150,

importance = FALSE, tree.err=FALSE)

**Table E1. Characteristics of study subjects in validation cohort by race and ethnicity at baseline, n (%) unless otherwise stated.**

| *Demographics and Lifestyle Characteristics* | Non-Hispanic White  N=141,329 | Non-Hispanic Black  N = 32,951 | Hispanic  N = 123,028 | Asian and Pacific Islander  N = 41,001 |
| --- | --- | --- | --- | --- |
| Age, mean (SD) | 64.66 (9.21) | 63.41 (9.23) | 61.53 (8.82) | 63.16 (9.10) |
| Female | 72,079 (51.00) | 19,220 (58.33) | 65,487 (53.23) | 22,867 (55.77) |
| Family History of Pancreatic Cancer | 3,271 (2.31) | 675 (2.05) | 2,068 (1.68) | 680 (1.66) |
| Tobacco Use  Ever  Never  Unknown | 56,190 (39.76)  84,078 (59,.49)  1,061 (0.75) | 12,519 (37.99)  20,265 (61.50)  167 (0.51) | 35,411 (28.78)  86,335 (70.18)  1,282 (1.04) | 10,086 (24.60)  30,546 (74.50)  369 (0.90) |
| Weight Defined by BMI (kg/m^2^)  Underweight (<18.5)  Normal Weight (18.5-24.9)  Overweight (25-29.9)  Obese (30+)  Unknown | 1,318 (0.93)  27,158 (19.22)  39,932 (28.25)  43,695 (30.92)  29,226 (20.68) | 227 (0.69)  3,927 (11.92)  8,013 (24.32)  13,828 (41.97)  6,956 (21.11) | 371 (0.30)  14,112 (11.47)  36,027 (29.28)  46,662 (37.93)  25,856 (21.02) | 678 (1.65)  13,338 (32.53)  12,094 (29.50)  5,058 (12.34)  9,833 (23.98) |
| Weight Change in 1 Year in lb., median (IQR)^a^ | -0.00 (-0.03, 0.02) | -0.00 (-0.03, 0.02) | -0.00 (-0.02, 0.02) | -0.00 (-0.02, 0.01) |
| *Lab Tests* |  |  |  |  |
| ALT, IU/L |  |  |  |  |
| N | 60,576 | 14,285 | 54,195 | 20,669 |
| Most recent, median (IQR) | 22.00 (17.00, 29.00) | 20.00 (16.00, 27.00) | 23.00 (18.00, 32.00) | 23.00 (18.00, 31.00) |
| Change in 1 year, median (IQR) | 0.00 (-4.00, 4.00) | 0.00 (-4.00, 4.00) | 0.00 (-5.00, 5.00) | 0.00 (-4.00, 4.00) |
| Change rate, median (IQR) | 0.00 (-0.01, 0.01) | 0.00 (-0.01, 0.01) | 0.00 (-0.01, 0.01) | 0.00 (-0.01, 0.01) |
| HgA1c, % |  |  |  |  |
| N | 68,452 | 18,288 | 69,201 | 25,747 |
| Most recent, median (IQR) | 5.70 (5.40, 6.30) | 6.00 (5.60, 6.90) | 6.10 (5.60, 7.20) | 6.00 (5.70, 6.80) |
| Change in 1 year, median (IQR) | 0.00 (-0.20, 0.20) | 0.00 (-0.30, 0.20) | 0.00 (-0.30, 0.20) | 0.00 (-0.20, 0.10) |
| HGB for Males, g/dL |  |  |  |  |
| N | 35,202 | 7,094 | 26,445 | 9,273 |
| Most recent, median (IQR) | 14.70 (13.60, 15.50) | 13.80 (12.60, 14.80) | 14.60 (13.60, 15.50) | 14.60 (13.50, 15.40) |
| HGB for Females, g/dL |  |  |  |  |
| N | 36,267 | 10,038 | 32,953 | 11,949 |
| Most recent, median (IQR) | 13.50 (12.60, 14.20) | 12.60 (11.70, 13.50) | 13.30 (12.40, 14.00) | 13.30 (12.50, 14.10) |
| HCT, L/L |  |  |  |  |
| N | 71,469 | 17,132 | 59,397 | 10,930 |
| Most recent, median (IQR) | 42.30 (39.40, 45.00) | 40.50 (37.40, 43.30) | 41.80 (38.90, 44.50) | 42.10 (39.20, 44.70) |
| Change in 1 year, median (IQR) | -0.20 (-2.10, 1.70) | 0.00 (-2.00, 1.90) | -0.10 (-2.00, 1.70) | -0.20 (-1.90, 1.50) |
| RBC, million/mm^3^ |  |  |  |  |
| N | 68,848 | 16,624 | 57,633 | 20,472 |
| Most recent, median (IQR) | 4.59 (4.24, 4.91) | 4.54 (4.15, 4.92) | 4.60 (4.27, 4.93) | 4.61 (4.27, 4.96) |
| Total Cholesterol, mg/dL |  |  |  |  |
| N | 58,401 | 13,201 | 51,475 | 20,955 |
| Most recent, median (IQR) | 179.00 (149.00, 212.00) | 176.00 (146.00, 207.00) | 178.00 (147.00, 210.00) | 178.00 (147.00, 212.00) |
| Platelets, count/L |  |  |  |  |
| N | 33,971 | 8,648 | 27,544 | 10,351 |
| Change rate in 1 year, median (IQR) | 0.00 (-0.06, 0.06) | 0.00 (-0.07, 0.07) | -0.00 (-0.06, 0.06) | 0.00 (-0.06, 0.05) |
| *Medical Conditions, 0-6 month prior* |  |  |  |  |
| Acute Pancreatitis | 246 (0.17) | 67 (0.20) | 237 (0.19) | 49 (0.12) |
| Chronic Pancreatitis | 175 (0.12) | 50 (0.15) | 88 (0.07) | 29 (0.07) |
| Benign Pancreatic Disease | 457 (0.32) | 75 (0.23) | 257 (0.21) | 131 (0.32) |
| *Medical Procedures, 0-6 month prior* |  |  |  |  |
| Surgical Procedures on esophagus | 1,027 (0.73) | 271 (0.82) | 836 (0.68) | 256 (0.62) |
| *Medications, 0-6 month prior* |  |  |  |  |
| Pancreatic Enzyme | 149 (0.11) | 28 (0.08) | 65 (0.05) | 23 (0.06) |
| *GI-Related Signs/Symptoms, 0-6 month prior* |  |  |  |  |
| Abdominal Pain | 10,180 (7.20) | 2,763 (8.39) | 12,412 (10.09) | 2,733 (6.67) |
| Constipation | 4,105 (2.90) | 1,257 (3.81) | 4,135 (3.36) | 957 (2.33) |
| Melena | 627 (0.44) | 176 (0.53) | 574 (0.47) | 183 (0.45) |

**Table E2. Characteristics and processing time for 53 weekly simulation batches.**

| **Batch start data** | **Batch size** | **PDAC cases** | **Incidence rate** | **Data extraction time (hr)** | **Imputation and model execution time (min)** | | |
| --- | --- | --- | --- | --- | --- | --- | --- |
|  |  |  |  |  | **SME** | **SMI** | **IFCE** |
| 01/01/2020 | 133,882 | 113 | 0.612 | 13.08 | 0.53 | 288.60 | 13.19 |
| 01/08/2020 | 197,105 | 165 | 0.596 | 13.01 | 0.73 | 398.41 | 19.67 |
| 01/15/2020 | 239,882 | 193 | 0.573 | 15.15 | 0.96 | 372.60 | 26.36 |
| 01/22/2020 | 231,643 | 186 | 0.572 | 14.92 | 0.87 | 374.49 | 26.91 |
| 01/29/2020 | 235,353 | 182 | 0.552 | 15.79 | 0.89 | 380.83 | 26.78 |
| 02/05/2020 | 233,098 | 190 | 0.580 | 15.43 | 0.88 | 464.96 | 28.86 |
| 02/12/2020 | 233,561 | 198 | 0.604 | 15.20 | 0.02 | 393.85 | 27.62 |
| 02/19/2020 | 225,451 | 170 | 0.538 | 14.29 | 0.97 | 311.79 | 23.96 |
| 02/26/2020 | 233,460 | 168 | 0.514 | 14.96 | 0.98 | 364.63 | 23.67 |
| 03/04/2020 | 233,902 | 202 | 0.615 | 14.97 | 0.02 | 413.05 | 25.61 |
| 03/11/2020 | 229,516 | 181 | 0.562 | 15.26 | 0.94 | 419.08 | 24.25 |
| 03/18/2020 | 201,192 | 164 | 0.584 | 12.87 | 0.83 | 473.95 | 21.02 |
| 03/25/2020 | 164,792 | 149 | 0.651 | 11.34 | 0.65 | 358.77 | 15.98 |
| 04/01/2020 | 147,694 | 127 | 0.621 | 11.69 | 0.61 | 327.37 | 13.77 |
| 04/08/2020 | 132,673 | 112 | 0.608 | 13.09 | 0.56 | 388.36 | 10.67 |
| 04/15/2020 | 124,959 | 115 | 0.664 | 10.43 | 0.57 | 472.24 | 11.20 |
| 04/22/2020 | 134,489 | 116 | 0.623 | 10.42 | 0.57 | 413.30 | 12.19 |
| 04/29/2020 | 143,317 | 126 | 0.636 | 14.19 | 0.60 | 318.15 | 11.29 |
| 05/06/2020 | 149,045 | 124 | 0.599 | 15.10 | 0.62 | 306.34 | 14.27 |
| 05/13/2020 | 150,257 | 143 | 0.686 | 15.07 | 0.64 | 398.30 | 14.26 |
| 05/20/2020 | 155,897 | 136 | 0.629 | 15.68 | 0.64 | 316.19 | 14.62 |
| 05/27/2020 | 141,735 | 142 | 0.723 | 14.72 | 0.60 | 301.94 | 13.12 |
| 06/03/2020 | 168,116 | 165 | 0.706 | 10.87 | 0.69 | 344.41 | 16.31 |
| 06/10/2020 | 171,240 | 156 | 0.656 | 11.47 | 0.69 | 396.46 | 16.93 |
| 06/17/2020 | 175,462 | 161 | 0.661 | 12.03 | 0.69 | 335.18 | 17.02 |
| 06/24/2020 | 181,739 | 164 | 0.651 | 12.26 | 0.72 | 386.21 | 17.58 |
| 07/01/2020 | 188,231 | 152 | 0.583 | 12.87 | 0.73 | 383.15 | 21.04 |
| 07/08/2020 | 166,079 | 143 | 0.619 | 11.01 | 0.65 | 385.13 | 16.35 |
| 07/15/2020 | 187,291 | 144 | 0.553 | 12.03 | 0.73 | 387.87 | 21.01 |
| 07/22/2020 | 186,557 | 130 | 0.502 | 12.22 | 0.73 | 315.00 | 19.35 |
| 07/29/2020 | 187,502 | 153 | 0.589 | 12.70 | 0.71 | 373.06 | 16.47 |
| 08/05/2020 | 185,850 | 152 | 0.588 | 12.17 | 0.73 | 425.38 | 19.74 |
| 08/12/2020 | 185,094 | 142 | 0.552 | 11.97 | 0.68 | 462.41 | 19.57 |
| 08/19/2020 | 187,757 | 140 | 0.537 | 12.61 | 0.73 | 388.68 | 19.90 |
| 08/26/2020 | 191,970 | 146 | 0.549 | 12.33 | 0.71 | 430.30 | 20.36 |
| 09/02/2020 | 198,986 | 158 | 0.572 | 12.63 | 0.76 | 303.38 | 20.52 |
| 09/09/2020 | 177,191 | 128 | 0.520 | 12.13 | 0.69 | 457.54 | 18.13 |
| 09/16/2020 | 204,244 | 126 | 0.445 | 13.46 | 0.73 | 317.09 | 20.89 |
| 09/23/2020 | 210,555 | 155 | 0.531 | 13.46 | 0.77 | 368.00 | 22.25 |
| 09/30/2020 | 215,097 | 168 | 0.564 | 14.03 | 0.88 | 429.07 | 21.07 |
| 10/07/2020 | 212,481 | 166 | 0.562 | 14.28 | 0.84 | 380.18 | 22.09 |
| 10/14/2020 | 212,089 | 158 | 0.536 | 13.55 | 0.82 | 461.51 | 22.26 |
| 10/21/2020 | 215,484 | 170 | 0.568 | 13.67 | 0.86 | 385.02 | 22.52 |
| 10/28/2020 | 213,544 | 160 | 0.540 | 13.49 | 0.78 | 315.71 | 22.41 |
| 11/04/2020 | 211,929 | 156 | 0.529 | 13.94 | 0.82 | 301.40 | 22.59 |
| 11/11/2020 | 213,052 | 166 | 0.560 | 14.00 | 0.77 | 451.16 | 22.70 |
| 11/18/2020 | 216,152 | 161 | 0.536 | 14.55 | 0.80 | 356.01 | 22.58 |
| 11/25/2020 | 207,878 | 151 | 0.523 | 13.78 | 0.83 | 443.95 | 21.70 |
| 12/02/2020 | 171,919 | 128 | 0.536 | 11.04 | 0.64 | 436.14 | 14.80 |
| 12/09/2020 | 214,600 | 151 | 0.506 | 13.62 | 0.83 | 446.76 | 23.24 |
| 12/16/2020 | 209,622 | 146 | 0.502 | 14.14 | 0.84 | 470.46 | 23.62 |
| 12/23/2020 | 195,811 | 149 | 0.550 | 13.29 | 0.71 | 389.21 | 18.20 |
| 12/30/2020 | 144,491 | 107 | 0.537 | 12.45 | 0.55 | 356.98 | 13.04 |

SME: Sub-models estimation; SMI: Stacked multiple imputation; IFCE: Imputation by fixed chained equations.

| **Batch start date** | **c-index** | **Top 2.5%** | | | | | **Top 1%** | | | | |
| --- | --- | --- | --- | --- | --- | --- | --- | --- | --- | --- | --- |
|  |  | **Patients screened** | **Cases detected** | **Number screened/ case** | **Sensitivity**  **(%)** | **PPV**  **(%)** | **Patients screened** | **Cases detected** | **Number screened/ case** | **Sensitivity**  **(%)** | **PPV**  **(%)** |
| 01/01/2020 | 0.88 | 2,874 | 38 | 76 | 33.6 | 1.3 | 1,151 | 24 | 48 | 21.2 | 2.1 |
| 01/08/2020 | 0.87 | 4,306 | 48 | 90 | 29.1 | 1.1 | 1,723 | 31 | 56 | 18.8 | 1.8 |
| 01/15/2020 | 0.85 | 5,250 | 52 | 101 | 26.9 | 1.0 | 2,100 | 37 | 57 | 19.2 | 1.8 |
| 01/22/2020 | 0.84 | 5,066 | 51 | 99 | 27.4 | 1.0 | 2,027 | 33 | 61 | 17.7 | 1.6 |
| 01/29/2020 | 0.84 | 5,144 | 53 | 97 | 29.1 | 1.0 | 2,058 | 25 | 83 | 13.7 | 1.2 |
| 02/05/2020 | 0.83 | 5,097 | 50 | 102 | 26.3 | 1.0 | 2,040 | 35 | 58 | 18.4 | 1.7 |
| 02/12/2020 | 0.85 | 5,116 | 56 | 92 | 28.3 | 1.1 | 2,046 | 41 | 50 | 20.7 | 2.0 |
| 02/19/2020 | 0.86 | 4,928 | 47 | 105 | 27.7 | 1.0 | 1,972 | 31 | 64 | 18.2 | 1.6 |
| 02/26/2020 | 0.86 | 5,106 | 46 | 111 | 27.4 | 0.9 | 2,043 | 35 | 58 | 20.8 | 1.7 |
| 03/04/2020 | 0.84 | 5,110 | 50 | 102 | 24.8 | 1.0 | 2,044 | 38 | 54 | 18.8 | 1.9 |
| 03/11/2020 | 0.84 | 5,019 | 44 | 114 | 24.3 | 0.9 | 2,008 | 35 | 57 | 19.3 | 1.7 |
| 03/18/2020 | 0.85 | 4,374 | 47 | 93 | 28.7 | 1.1 | 1,750 | 26 | 67 | 15.9 | 1.5 |
| 03/25/2020 | 0.87 | 3,552 | 48 | 74 | 32.2 | 1.4 | 1,423 | 28 | 51 | 18.8 | 2.0 |
| 04/01/2020 | 0.87 | 3,174 | 38 | 83 | 29.9 | 1.2 | 1,270 | 19 | 67 | 15.0 | 1.5 |
| 04/08/2020 | 0.86 | 2,847 | 31 | 92 | 27.7 | 1.1 | 1,139 | 24 | 47 | 21.4 | 2.1 |
| 04/15/2020 | 0.87 | 2,675 | 30 | 89 | 26.1 | 1.1 | 1,071 | 19 | 56 | 16.5 | 1.8 |
| 04/22/2020 | 0.87 | 2,882 | 35 | 83 | 30.2 | 1.2 | 1,153 | 27 | 43 | 23.3 | 2.3 |
| 04/29/2020 | 0.87 | 3,070 | 31 | 99 | 24.6 | 1.0 | 1,229 | 22 | 56 | 17.5 | 1.8 |
| 05/06/2020 | 0.88 | 3,265 | 39 | 84 | 31.5 | 1.2 | 1,281 | 21 | 61 | 16.9 | 1.6 |
| 05/13/2020 | 0.85 | 3,230 | 42 | 77 | 29.4 | 1.3 | 1,297 | 27 | 48 | 18.9 | 2.1 |
| 05/20/2020 | 0.87 | 3,357 | 44 | 76 | 32.4 | 1.3 | 1,343 | 30 | 45 | 22.1 | 2.2 |
| 05/27/2020 | 0.86 | 3,051 | 41 | 75 | 28.9 | 1.3 | 1,221 | 30 | 41 | 21.1 | 2.5 |
| 06/03/2020 | 0.86 | 3,626 | 56 | 65 | 33.9 | 1.5 | 1,451 | 33 | 44 | 20.0 | 2.3 |
| 06/10/2020 | 0.85 | 3,695 | 44 | 84 | 28.2 | 1.2 | 1,478 | 33 | 45 | 21.2 | 2.2 |
| 06/17/2020 | 0.85 | 3,793 | 48 | 79 | 29.8 | 1.3 | 1,517 | 30 | 51 | 18.6 | 2.0 |
| 06/24/2020 | 0.84 | 3,930 | 44 | 89 | 26.8 | 1.1 | 1,677 | 32 | 52 | 19.5 | 1.9 |
| 07/01/2020 | 0.86 | 4,075 | 47 | 87 | 30.9 | 1.2 | 1,639 | 31 | 53 | 20.4 | 1.9 |
| 07/08/2020 | 0.86 | 3,570 | 43 | 83 | 30.1 | 1.2 | 1,428 | 31 | 46 | 21.7 | 2.2 |
| 07/15/2020 | 0.86 | 4,028 | 39 | 103 | 27.1 | 1.0 | 1,612 | 29 | 56 | 20.1 | 1.8 |
| 07/22/2020 | 0.86 | 4,006 | 43 | 93 | 33.1 | 1.1 | 1,603 | 32 | 50 | 24.6 | 2.0 |
| 07/29/2020 | 0.86 | 4,025 | 46 | 88 | 30.1 | 1.1 | 1,612 | 29 | 56 | 19.0 | 1.8 |
| 08/05/2020 | 0.85 | 3,992 | 42 | 95 | 27.6 | 1.1 | 1,598 | 29 | 55 | 19.1 | 1.8 |
| 08/12/2020 | 0.85 | 3,975 | 38 | 104 | 26.8 | 1.0 | 1,590 | 24 | 66 | 16.9 | 1.5 |
| 08/19/2020 | 0.86 | 4,031 | 41 | 98 | 29.3 | 1.0 | 1,612 | 26 | 62 | 18.6 | 1.6 |
| 08/26/2020 | 0.86 | 4,117 | 43 | 96 | 29.5 | 1.0 | 1,647 | 27 | 61 | 18.5 | 1.6 |
| 09/02/2020 | 0.86 | 4,271 | 37 | 115 | 23.4 | 0.9 | 1,709 | 27 | 63 | 17.1 | 1.6 |
| 09/09/2020 | 0.87 | 3,808 | 40 | 95 | 31.3 | 1.1 | 1,524 | 24 | 64 | 18.8 | 1.6 |
| 09/16/2020 | 0.86 | 4,395 | 47 | 93 | 37.3 | 1.1 | 1,758 | 25 | 70 | 19.8 | 1.4 |
| 09/23/2020 | 0.84 | 4,533 | 43 | 105 | 27.7 | 1.0 | 1,813 | 23 | 79 | 14.8 | 1.3 |
| 09/30/2020 | 0.84 | 4,634 | 47 | 99 | 28.0 | 1.0 | 1,854 | 28 | 66 | 16.7 | 1.5 |
| 10/07/2020 | 0.85 | 4,583 | 46 | 100 | 27.7 | 1.0 | 1,834 | 28 | 65 | 16.9 | 1.5 |
| 10/14/2020 | 0.85 | 4,577 | 43 | 106 | 27.2 | 0.9 | 1,831 | 27 | 68 | 17.1 | 1.5 |
| 10/21/2020 | 0.84 | 4,660 | 42 | 111 | 24.7 | 0.9 | 1,864 | 28 | 67 | 16.5 | 1.5 |
| 10/28/2020 | 0.85 | 4,615 | 42 | 110 | 26.3 | 0.9 | 1,847 | 26 | 71 | 16.3 | 1.4 |
| 11/04/2020 | 0.84 | 4,591 | 44 | 104 | 28.2 | 1.0 | 1,836 | 32 | 57 | 20.5 | 1.7 |
| 11/11/2020 | 0.85 | 4,616 | 44 | 105 | 26.5 | 1.0 | 1,847 | 24 | 77 | 14.5 | 1.3 |
| 11/18/2020 | 0.85 | 4,684 | 47 | 99 | 24.8 | 1.0 | 1,875 | 28 | 67 | 17.4 | 1.5 |
| 11/25/2020 | 0.84 | 4,500 | 40 | 112 | 26.5 | 0.9 | 1,800 | 23 | 78 | 15.2 | 1.3 |
| 12/02/2020 | 0.84 | 3,715 | 39 | 95 | 30.5 | 1.1 | 1,486 | 25 | 60 | 19.5 | 1.7 |
| 12/09/2020 | 0.85 | 4,646 | 43 | 108 | 28.5 | 0.9 | 1,859 | 23 | 81 | 15.2 | 1.2 |
| 12/16/2020 | 0.85 | 4,535 | 36 | 127 | 24.7 | 0.8 | 1,813 | 18 | 101 | 12.3 | 1.0 |
| 12/23/2020 | 0.86 | 4,233 | 41 | 103 | 27.5 | 1.0 | 1,695 | 25 | 68 | 16.8 | 1.5 |
| 12/30/2020 | 0.88 | 3,115 | 40 | 78 | 37.4 | 1.3 | 1,246 | 19 | 66 | 17.8 | 1.5 |

**Table E3a. Batch-level predictive performance using Sub-Model Estimation (SME) for imputation.**

**Table E3b. Batch-level predictive performance using Stacking Method Imputation (SMI) for imputation.**

| **Batch start date** | **c-index** | **Top 2.5%** | | | | | **Top 1%** | | | | |
| --- | --- | --- | --- | --- | --- | --- | --- | --- | --- | --- | --- |
|  |  | **Patients screened** | **Cases detected** | **Number screened/ case** | **Sensitivity**  **(%)** | **PPV**  **(%)** | **Patients screened** | **Cases detected** | **Number screened/ case** | **Sensitivity (%)** | **PPV**  **(%)** |
| 01/01/2020 | 0.80 | 3,347 | 28 | 119 | 24.8 | 0.8 | 1,338 | 15 | 89 | 13.3 | 1.1 |
| 01/08/2020 | 0.78 | 4,928 | 28 | 175 | 17.0 | 0.6 | 1,971 | 19 | 104 | 11.5 | 1.0 |
| 01/15/2020 | 0.78 | 5,998 | 35 | 172 | 18.1 | 0.6 | 2,398 | 20 | 120 | 10.4 | 0.8 |
| 01/22/2020 | 0.77 | 5,792 | 33 | 175 | 17.7 | 0.6 | 2,316 | 24 | 95 | 11.8 | 1.1 |
| 01/29/2020 | 0.76 | 5,884 | 31 | 189 | 17.0 | 0.5 | 2,353 | 19 | 123 | 10.4 | 0.8 |
| 02/05/2020 | 0.77 | 5,828 | 40 | 145 | 21.1 | 0.7 | 2,330 | 21 | 111 | 11.1 | 0.9 |
| 02/12/2020 | 0.78 | 5,116 | 38 | 135 | 19.2 | 0.7 | 2,046 | 25 | 82 | 12.6 | 1.2 |
| 02/19/2020 | 0.79 | 4,932 | 39 | 127 | 22.9 | 0.8 | 1,973 | 18 | 110 | 10.6 | 0.9 |
| 02/26/2020 | 0.78 | 5,106 | 37 | 139 | 22.0 | 0.7 | 2,043 | 21 | 97 | 12.5 | 1.0 |
| 03/04/2020 | 0.78 | 5,110 | 42 | 122 | 20.8 | 0.8 | 2,045 | 21 | 97 | 10.4 | 1.0 |
| 03/11/2020 | 0.77 | 5,017 | 38 | 132 | 21.0 | 0.8 | 2,008 | 25 | 80 | 13.8 | 1.3 |
| 03/18/2020 | 0.80 | 4,374 | 39 | 112 | 23.8 | 0.9 | 1,750 | 25 | 70 | 15.2 | 1.4 |
| 03/25/2020 | 0.78 | 3,552 | 27 | 132 | 18.1 | 0.8 | 1,421 | 17 | 83 | 11.4 | 1.2 |
| 04/01/2020 | 0.77 | 3,175 | 23 | 139 | 18.1 | 0.7 | 1,271 | 14 | 91 | 11.0 | 1.1 |
| 04/08/2020 | 0.76 | 2,847 | 15 | 189 | 13.4 | 0.5 | 1,139 | 12 | 95 | 10.7 | 1.1 |
| 04/15/2020 | 0.74 | 2,677 | 21 | 128 | 18.3 | 0.8 | 1,070 | 13 | 83 | 11.3 | 1.2 |
| 04/22/2020 | 0.77 | 2,882 | 19 | 152 | 16.4 | 0.7 | 1,153 | 15 | 77 | 12.9 | 1.3 |
| 04/29/2020 | 0.79 | 3,070 | 27 | 114 | 21.4 | 0.9 | 1,228 | 16 | 77 | 12.7 | 1.3 |
| 05/06/2020 | 0.74 | 3,203 | 22 | 145 | 17.7 | 0.7 | 1,281 | 15 | 85 | 12.1 | 1.2 |
| 05/13/2020 | 0.72 | 3,229 | 25 | 130 | 17.5 | 0.8 | 1,292 | 16 | 81 | 11.2 | 1.2 |
| 05/20/2020 | 0.77 | 3,360 | 23 | 147 | 16.9 | 0.7 | 1,344 | 15 | 89 | 11.0 | 1.1 |
| 05/27/2020 | 0.74 | 3,054 | 17 | 179 | 12.0 | 0.6 | 1,221 | 13 | 94 | 9.2 | 1.1 |
| 06/03/2020 | 0.77 | 3,628 | 31 | 118 | 18.8 | 0.9 | 1,451 | 21 | 69 | 12.7 | 1.5 |
| 06/10/2020 | 0.76 | 3,695 | 25 | 147 | 16.0 | 0.7 | 1,478 | 15 | 99 | 9.6 | 1.0 |
| 06/17/2020 | 0.73 | 3,792 | 23 | 164 | 14.3 | 0.6 | 1,517 | 15 | 101 | 9.3 | 1.0 |
| 06/24/2020 | 0.74 | 3,932 | 27 | 145 | 16.5 | 0.7 | 1,572 | 17 | 93 | 10.4 | 1.1 |
| 07/01/2020 | 0.75 | 4,075 | 27 | 152 | 17.8 | 0.7 | 1,630 | 19 | 85 | 12.5 | 1.2 |
| 07/08/2020 | 0.75 | 3,571 | 27 | 132 | 18.9 | 0.8 | 1,428 | 19 | 75 | 13.3 | 1.3 |
| 07/15/2020 | 0.74 | 4,028 | 25 | 161 | 17.4 | 0.6 | 1,612 | 17 | 95 | 11.8 | 1.1 |
| 07/22/2020 | 0.76 | 4,006 | 24 | 167 | 18.5 | 0.6 | 1,603 | 17 | 94 | 13.1 | 1.1 |
| 07/29/2020 | 0.75 | 4,028 | 28 | 143 | 18.3 | 0.7 | 1,610 | 18 | 89 | 11.8 | 1.1 |
| 08/05/2020 | 0.72 | 3,992 | 25 | 159 | 16.5 | 0.6 | 1,597 | 12 | 133 | 7.9 | 0.8 |
| 08/12/2020 | 0.74 | 3,975 | 20 | 200 | 14.1 | 0.5 | 1,590 | 11 | 145 | 7.8 | 0.7 |
| 08/19/2020 | 0.76 | 4,030 | 23 | 175 | 16.4 | 0.6 | 1,613 | 13 | 123 | 9.3 | 0.8 |
| 08/26/2020 | 0.74 | 4,117 | 26 | 159 | 17.8 | 0.6 | 1,647 | 15 | 110 | 10.3 | 0.9 |
| 09/02/2020 | 0.76 | 4,271 | 22 | 192 | 13.9 | 0.5 | 1,710 | 13 | 132 | 8.2 | 0.8 |
| 09/09/2020 | 0.77 | 3,808 | 20 | 189 | 15.6 | 0.5 | 1,524 | 14 | 109 | 10.9 | 0.9 |
| 09/16/2020 | 0.75 | 4,395 | 18 | 244 | 14.3 | 0.4 | 1,758 | 12 | 147 | 9.5 | 0.7 |
| 09/23/2020 | 0.73 | 4,535 | 20 | 227 | 12.9 | 0.4 | 1,817 | 13 | 139 | 8.4 | 0.7 |
| 09/30/2020 | 0.71 | 4,649 | 20 | 233 | 11.9 | 0.4 | 1,854 | 13 | 143 | 7.7 | 0.7 |
| 10/07/2020 | 0.77 | 4,583 | 31 | 147 | 18.7 | 0.7 | 1,834 | 20 | 92 | 12.1 | 1.1 |
| 10/14/2020 | 0.74 | 4,577 | 26 | 175 | 16.5 | 0.6 | 1,831 | 15 | 122 | 9.5 | 0.8 |
| 10/21/2020 | 0.75 | 4,660 | 24 | 192 | 14.1 | 0.5 | 1,864 | 15 | 125 | 8.8 | 0.8 |
| 10/28/2020 | 0.73 | 4,615 | 25 | 185 | 15.6 | 0.5 | 1,850 | 17 | 109 | 10.6 | 0.9 |
| 11/04/2020 | 0.72 | 4,590 | 22 | 208 | 14.1 | 0.5 | 1,837 | 16 | 115 | 10.3 | 0.9 |
| 11/11/2020 | 0.76 | 4,617 | 32 | 145 | 19.3 | 0.7 | 1,849 | 23 | 81 | 13.9 | 1.2 |
| 11/18/2020 | 0.73 | 4,684 | 24 | 196 | 14.9 | 0.5 | 1,874 | 15 | 125 | 9.3 | 0.8 |
| 11/25/2020 | 0.74 | 4,500 | 21 | 213 | 13.9 | 0.5 | 1,800 | 15 | 120 | 9.9 | 0.8 |
| 12/02/2020 | 0.76 | 3,715 | 20 | 185 | 15.6 | 0.5 | 1,486 | 13 | 115 | 10.2 | 0.9 |
| 12/09/2020 | 0.74 | 4,646 | 24 | 192 | 15.9 | 0.5 | 1,860 | 14 | 133 | 9.3 | 0.8 |
| 12/16/2020 | 0.75 | 4,531 | 25 | 182 | 17.1 | 0.6 | 1,813 | 15 | 120 | 10.3 | 0.8 |
| 12/23/2020 | 0.74 | 4,233 | 23 | 185 | 15.4 | 0.5 | 1,694 | 15 | 112 | 10.1 | 0.9 |
| 12/30/2020 | 0.77 | 3,114 | 21 | 149 | 19.6 | 0.7 | 1,247 | 12 | 104 | 11.2 | 1.0 |

**Table E3c. Batch-level predictive performance using Imputation by Fixed Chained Equations (IFCE) for imputation.**

| **Batch start date** | **c-index** | **Top 2.5%** | | | | | **Top 1%** | | | | |
| --- | --- | --- | --- | --- | --- | --- | --- | --- | --- | --- | --- |
|  |  | **Patients screened** | **Cases detected** | **Number screened/ case** | **Sensitivity**  **(%)** | **PPV**  **(%)** | **Patients screened** | **Cases detected** | **Number screened/ case** | **Sensitivity**  **(%)** | **PPV**  **(%)** |
| 01/01/2020 | 0.80 | 2,874 | 27 | 106 | 23.9 | 0.9 | 1,150 | 17 | 68 | 15.0 | 1.5 |
| 01/08/2020 | 0.80 | 4,306 | 41 | 105 | 24.9 | 1.0 | 1,723 | 24 | 72 | 14.6 | 1.4 |
| 01/15/2020 | 0.79 | 5,250 | 55 | 95 | 28.5 | 1.1 | 2,100 | 31 | 68 | 16.1 | 1.5 |
| 01/22/2020 | 0.78 | 5,066 | 40 | 127 | 21.5 | 0.8 | 2,027 | 24 | 85 | 12.9 | 1.2 |
| 01/29/2020 | 0.77 | 5,144 | 35 | 147 | 19.2 | 0.7 | 2,058 | 22 | 93 | 12.1 | 1.1 |
| 02/05/2020 | 0.78 | 5,097 | 44 | 116 | 23.2 | 0.9 | 2,039 | 29 | 70 | 15.3 | 1.4 |
| 02/12/2020 | 0.81 | 5,115 | 56 | 92 | 28.3 | 1.1 | 2,046 | 34 | 60 | 17.2 | 1.7 |
| 02/19/2020 | 0.81 | 4,928 | 49 | 101 | 28.8 | 1.0 | 1,972 | 36 | 55 | 21.2 | 1.8 |
| 02/26/2020 | 0.81 | 5,106 | 43 | 119 | 25.6 | 0.8 | 2,043 | 28 | 73 | 16.7 | 1.4 |
| 03/04/2020 | 0.80 | 5,110 | 54 | 94 | 26.7 | 1.1 | 2,044 | 39 | 52 | 19.3 | 1.9 |
| 03/11/2020 | 0.78 | 5,017 | 40 | 125 | 22.1 | 0.8 | 2,007 | 24 | 83 | 13.3 | 1.2 |
| 03/18/2020 | 0.78 | 4,374 | 35 | 125 | 21.3 | 0.8 | 1,750 | 20 | 88 | 12.2 | 1.1 |
| 03/25/2020 | 0.80 | 3,552 | 31 | 115 | 20.8 | 0.9 | 1,421 | 20 | 71 | 13.4 | 1.4 |
| 04/01/2020 | 0.78 | 3,174 | 26 | 122 | 20.5 | 0.8 | 1,270 | 19 | 67 | 15.0 | 1.5 |
| 04/08/2020 | 0.80 | 2,847 | 33 | 86 | 29.5 | 1.2 | 1,139 | 20 | 57 | 17.9 | 1.8 |
| 04/15/2020 | 0.80 | 2,675 | 29 | 93 | 25.2 | 1.1 | 1,070 | 25 | 43 | 21.7 | 2.3 |
| 04/22/2020 | 0.76 | 2,881 | 23 | 125 | 19.8 | 0.8 | 1,153 | 15 | 77 | 12.9 | 1.3 |
| 04/29/2020 | 0.77 | 3,070 | 22 | 139 | 17.5 | 0.7 | 1,228 | 13 | 94 | 10.3 | 1.1 |
| 05/06/2020 | 0.76 | 3,203 | 26 | 123 | 21.0 | 0.8 | 1,281 | 20 | 64 | 16.1 | 1.6 |
| 05/13/2020 | 0.77 | 3,229 | 26 | 123 | 18.2 | 0.8 | 1,292 | 21 | 61 | 14.7 | 1.6 |
| 05/20/2020 | 0.82 | 3,356 | 38 | 88 | 27.9 | 1.1 | 1,343 | 27 | 50 | 19.9 | 2.0 |
| 05/27/2020 | 0.82 | 3,051 | 45 | 68 | 31.7 | 1.5 | 1,221 | 35 | 35 | 24.7 | 2.9 |
| 06/03/2020 | 0.81 | 3,626 | 44 | 83 | 26.7 | 1.2 | 1,451 | 26 | 56 | 15.8 | 1.8 |
| 06/10/2020 | 0.79 | 3,695 | 39 | 94 | 25.0 | 1.1 | 1,478 | 25 | 59 | 16.0 | 1.7 |
| 06/17/2020 | 0.77 | 3,792 | 36 | 105 | 22.4 | 1.0 | 1,517 | 27 | 56 | 16.8 | 1.8 |
| 06/24/2020 | 0.82 | 3,930 | 55 | 71 | 33.5 | 1.4 | 1,572 | 35 | 45 | 21.3 | 2.2 |
| 07/01/2020 | 0.80 | 4,075 | 44 | 93 | 29.0 | 1.1 | 1,630 | 33 | 50 | 21.7 | 2.0 |
| 07/08/2020 | 0.80 | 3,570 | 44 | 81 | 30.8 | 1.2 | 1,428 | 29 | 49 | 20.3 | 2.0 |
| 07/15/2020 | 0.82 | 4,028 | 49 | 82 | 34.0 | 1.2 | 1,612 | 30 | 54 | 20.8 | 1.9 |
| 07/22/2020 | 0.85 | 4,006 | 55 | 73 | 42.3 | 1.4 | 1,603 | 39 | 41 | 30.0 | 2.4 |
| 07/29/2020 | 0.80 | 4,025 | 47 | 85 | 30.7 | 1.2 | 1,610 | 34 | 47 | 22.2 | 2.1 |
| 08/05/2020 | 0.77 | 3,992 | 33 | 120 | 21.7 | 0.8 | 1,597 | 26 | 61 | 17.1 | 1.6 |
| 08/12/2020 | 0.75 | 3,975 | 27 | 147 | 19.0 | 0.7 | 1,590 | 17 | 93 | 12.0 | 1.1 |
| 08/19/2020 | 0.77 | 4,030 | 26 | 154 | 18.6 | 0.7 | 1,612 | 16 | 101 | 11.4 | 1.0 |
| 08/26/2020 | 0.77 | 4,117 | 36 | 115 | 24.7 | 0.9 | 1,647 | 21 | 78 | 14.4 | 1.3 |
| 09/02/2020 | 0.77 | 4,271 | 33 | 130 | 20.9 | 0.8 | 1,709 | 21 | 81 | 13.3 | 1.2 |
| 09/09/2020 | 0.79 | 3,808 | 32 | 119 | 25.0 | 0.8 | 1,524 | 17 | 89 | 13.3 | 1.1 |
| 09/16/2020 | 0.79 | 4,395 | 26 | 169 | 20.6 | 0.6 | 1,758 | 18 | 98 | 14.3 | 1.0 |
| 09/23/2020 | 0.74 | 4,533 | 26 | 175 | 16.8 | 0.6 | 1,813 | 14 | 130 | 9.0 | 0.8 |
| 09/30/2020 | 0.75 | 4,634 | 30 | 154 | 17.9 | 0.7 | 1,854 | 16 | 116 | 9.5 | 0.9 |
| 10/07/2020 | 0.80 | 4,583 | 40 | 115 | 24.1 | 0.9 | 1,834 | 26 | 70 | 15.7 | 1.4 |
| 10/14/2020 | 0.78 | 4,577 | 39 | 118 | 24.7 | 0.9 | 1,831 | 29 | 63 | 18.4 | 1.6 |
| 10/21/2020 | 0.79 | 4,660 | 41 | 114 | 24.1 | 0.9 | 1,864 | 25 | 75 | 14.7 | 1.3 |
| 10/28/2020 | 0.77 | 4,615 | 33 | 139 | 20.6 | 0.7 | 1,846 | 21 | 88 | 13.1 | 1.1 |
| 11/04/2020 | 0.74 | 4,590 | 25 | 185 | 16.0 | 0.5 | 1,836 | 17 | 108 | 10.9 | 0.9 |
| 11/11/2020 | 0.79 | 4,616 | 40 | 115 | 24.1 | 0.9 | 1,847 | 24 | 77 | 14.5 | 1.3 |
| 11/18/2020 | 0.79 | 4,684 | 37 | 127 | 21.7 | 0.8 | 1,874 | 25 | 75 | 15.5 | 1.3 |
| 11/25/2020 | 0.78 | 4,500 | 32 | 141 | 21.2 | 0.7 | 1,800 | 19 | 94 | 12.6 | 1.1 |
| 12/02/2020 | 0.78 | 3,715 | 27 | 137 | 21.1 | 0.7 | 1,486 | 13 | 115 | 10.2 | 0.9 |
| 12/09/2020 | 0.79 | 4,646 | 42 | 111 | 27.8 | 0.9 | 1,859 | 24 | 78 | 15.9 | 1.3 |
| 12/16/2020 | 0.79 | 4,531 | 32 | 141 | 21.9 | 0.7 | 1,813 | 15 | 120 | 10.3 | 0.8 |
| 12/23/2020 | 0.80 | 4,233 | 38 | 111 | 25.5 | 0.9 | 1,694 | 28 | 61 | 18.8 | 1.7 |
| 12/30/2020 | 0.81 | 3,114 | 30 | 104 | 28.0 | 1.0 | 1,246 | 16 | 78 | 15.0 | 1.3 |
